# An improved model for target detection and pose estimation of a teleoperation power manipulator

**DOI:** 10.3389/fnbot.2023.1193823

**Published:** 2023-08-02

**Authors:** Li Xie, Jiale Huang, Yutian Li, Jianwen Guo

**Affiliations:** ^1^School of Mechanical Engineering, Dongguan University of Technology, Dongguan, China; ^2^School of Electromechanical Engineering, Guangdong University of Technology, Guangzhou, China

**Keywords:** teleoperation power manipulator, camera, target detection, pose estimation, deep learning

## Abstract

**Introduction:**

A hot cell is generally deployed with a teleoperation power manipulator to complete tests, operations, and maintenance. The position and pose of the manipulator are mostly acquired through radiation-resistant video cameras arranged in the hot cell. In this paper, deep learning-based target detection technology is used to establish an experimental platform to test the methods for target detection and pose estimation of teleoperation power manipulators using two cameras.

**Methods:**

In view of the fact that a complex environment affects the precision of manipulator pose estimation, the dilated-fully convolutional one-stage object detection (dilated-FCOS) teleoperation power manipulator target detection algorithm is proposed based on the scale of the teleoperation power manipulator. Model pruning is used to improve the real-time performance of the dilated-FCOS teleoperation power manipulator target detection model. To improve the detection speed for the key points of the teleoperation power manipulator, the keypoint detection precision and model inference speed of different lightweight backbone networks were tested based on the SimpleBaseline algorithm. MobileNetv1 was selected as the backbone network to perform channel compression and pose distillation on the upsampling module so as to further optimize the inference speed of the model.

**Results and discussion:**

Compared with the original model, the proposed model was experimentally proven to reach basically the same precision within a shorter inference time (only 58% of that of the original model). The experimental results show that the compressed model basically retains the precision of the original model and that its inference time is 48% of that of the original model.

## 1. Introduction

Hot cells in nuclear power plants and high-energy physics devices are shielded from radiation (Zheng et al., 2015; Zhang et al., 2022), and they play a crucial role in testing, operation, and maintenance activities. To facilitate tasks such as inspection, assembly, disassembly, transportation, and part repair, hot cells are equipped with either a master-slave manipulator or a teleoperation power manipulator (Pezhman and Saeed, 2011; Assem et al., 2014; Zhang et al., 2021). These manipulators are necessary to mitigate the harmful effects of radiation on humans. To assist the teleoperator, the teleoperation power manipulator relies on sensing technologies, including visual sensing (Maruyama et al., 2014) and force sensing (Oosterhout et al., 2012), to gather information about the operation area.

In hot cells, where the radiation environment limits the use of certain sensors, radiation-resistant cameras are commonly installed to capture on-site images and transmit them to operators via the network. To regularly replace single modules in a tokamak vessel, Qiu et al. (2016) used a hand–eye coordination method to ensure the consistency between the operator's hand movement and the manipulator's end effector movement. Ribeiro et al. (2020) designed a hand–eye camera system for the acquisition of key information in the operating environment. Lionel et al. (2018) introduced the virtual reality technology in the assembly and tooling design of the tokamak diverter to assist teleoperators and successfully achieved assembly with a gap of <1 mm. Ferreira et al. (2012) designed a localization system based on cameras to accurately estimate the position and direction of CPRH by capturing video streams for the implementation of an augmented reality system. Liu et al. (2020) proposed the vision-based breakpoint detection algorithm and successfully identified and captured tiles that had fallen onto the diverter by employing the watershed segmentation algorithm.

Most of the information about the teleoperation power manipulator's position and pose comes from radiation-resistant cameras in the hot cell. The operator's teleoperation efficiency is impacted by the limited visual information provided by this method of observation solely by human eyes through cameras. The application of technologies such as virtual reality (VR) or augmented reality (AR) can integrate the information of cameras into the operation platform of VR or AR, which is conducive to improving the operation efficiency (Qiu et al., 2016; Lionel et al., 2018; Ribeiro et al., 2020). However, obtaining the teleoperation power manipulator's position and pose from the photographs is one of the issues that need to be resolved in the hot cell.

The deep learning-based pose estimation algorithm can quickly distinguish poses from RGB images and achieve satisfactory estimation results. Kehl et al. (2017) proposed a direct regression-based 6D pose estimation method to achieve end-to-end 6D pose estimation. DeePose (Toshev and Szegedy, 2014) applied a convolutional neural network (CNN) to human pose estimation for the first time and achieved higher precision than traditional methods. Pose coordinate regression-based algorithms, on the other hand, only constrain the pose coordinates with the mean square error and ignore the supervision of the spatial information of the key points, making it difficult to further improve their regression precision. Wei et al. (2016) proposed a sequential architecture composed of convolutional networks to predict the locations of the key points and introduced the key points heatmap as the input of the next stage, which provides rich spatial information for the subsequent network layer and improves the robustness of the algorithm. Sun et al. (2017) proposed HRNet, which is composed of multi-resolution subnetworks connected in parallel and achieved the best pose estimation results on the COCO dataset in 2019. Mišeikis et al. (2018a,b) proposed a multi-objective CNN, which uses 2D images to estimate the 3D positions of the key points and used transfer learning techniques to adapt the CNN trained to estimate the poses of UR robots to Kuka robots. Heindl et al. (2019) proposed a multi-robot pose estimation method based on a recurrent neural network, which uses 2D images as input and simultaneously infers the number of robots in the scene, the joint locations, and the sparse depth maps around the joint locations, demonstrating high generalizability to the real-world images. Ning et al. (2020) presents a real-time 3D face-alignment method that uses an encoder-decoder network with an efficient deconvolution layer which has low prediction errors with real-time applicability. Wu et al. (2022) presents an age-compensated makeup transformation framework based on homology continuity, and the experimental results show that the framework outperforms existing methods.

The technical conditions for the pose estimation of teleoperation power manipulators are provided by the aforementioned studies. In this paper, target detection and pose estimation of teleoperation power manipulators are designed based on deep learning, obtaining the teleoperation power manipulator's position and pose by two cameras in the hot cell, which is few studied in this field at present. A dilated-fully convolutional one-stage object detection (dilated-FCOS) target detection algorithm for teleoperation power manipulators is suggested in accordance with its scale. For teleoperation power manipulators, a keypoint detection algorithm based on SimpleBaseline has been developed. This algorithm reduces the model's inference time while maintaining model precision. Through teleoperation power manipulator pose estimation experiments, an experimental platform for teleoperation power manipulator operation is established to confirm the methods' viability and efficacy.

The following is the layout of the remainder of the paper: the construction of the experimental platform and the production of the experimental data are both covered in detail in Section 2; the proposed dilated-FCOS teleoperation power manipulator target detection method is presented in Sections 3; the keypoint detection method is in the Section 4; experiments and discussion are the main focus of Section 5; summary of this work and suggestions for future research are presented in Section 6.

## 2. Experimental platform and experimental data

### 2.1. Construction of the experimental platform

**Figure 1:**
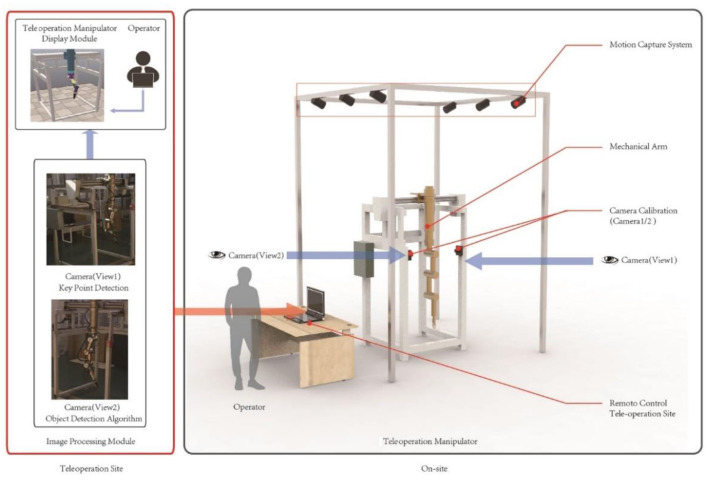
Experimental platform.

The experimental platform (Figure 1) consists of several components: a teleoperation power manipulator, a camera system with two cameras, a motion capture system, an image processing module, and a teleoperation power manipulator display module. The camera system captures real-time operational images of the teleoperation power manipulator, while the image processing module detects targets and estimates the pose of the manipulator. The updated pose information is then inputted into the teleoperation power manipulator display module to adjust its position accordingly.

**Figure 2:**
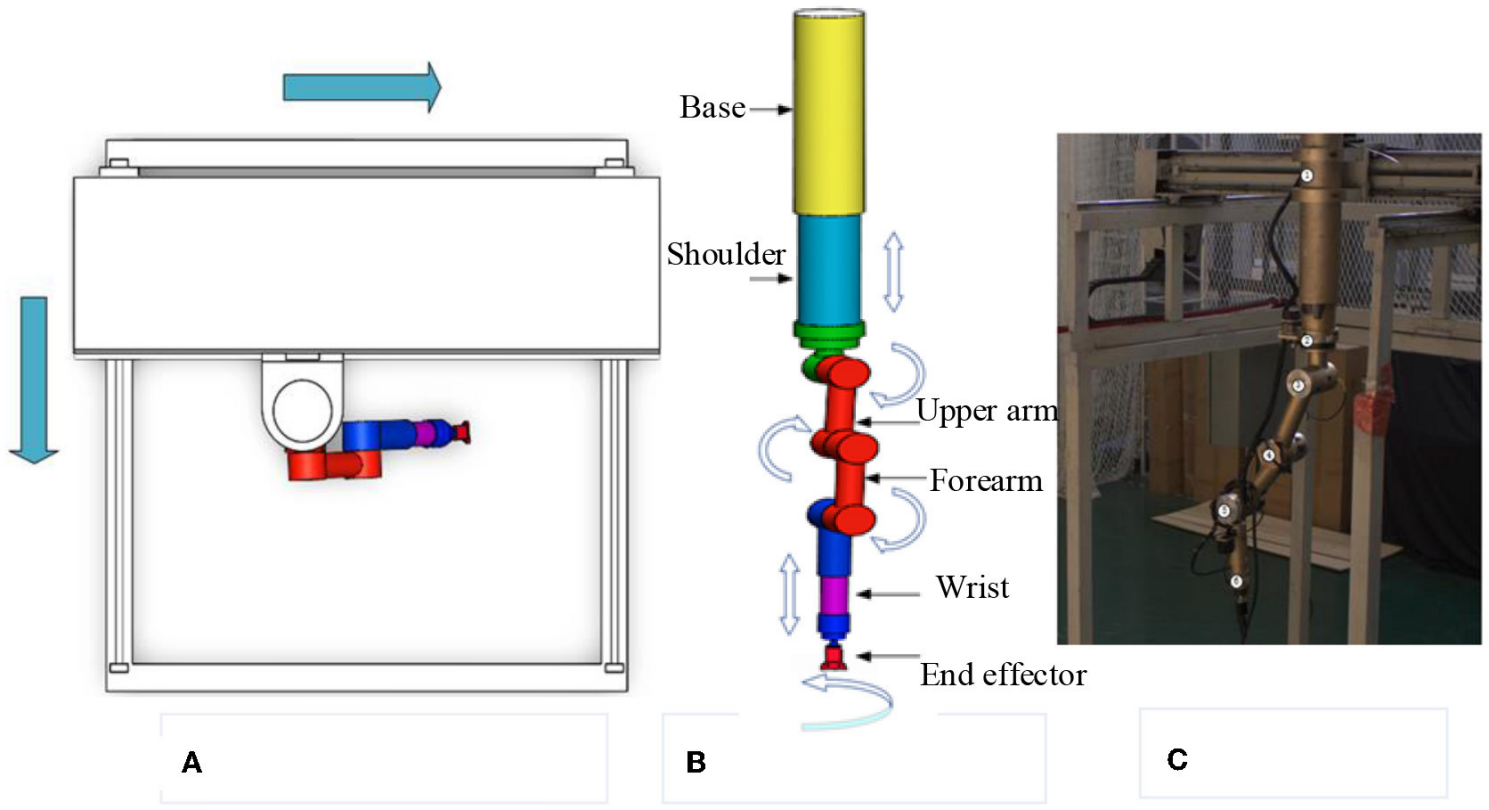
Model of the teleoperation power manipulator. **(A)** Mobile platform model. **(B)** Manipulator model. **(C)** Real manipulator.

(1) Teleoperation power manipulator. Figure 2C depicts the teleoperation power manipulator for teleoperation. It is configured with eight degrees of freedom, consisting of four rotational and four translational degrees of freedom. The mobile platform, depicted in Figure 2A in two dimensions, allows the teleoperation power manipulator to move forwards and backwards to reach the desired operational position. Figure 2B presents the 3D model of the teleoperation power manipulator, which includes a base, a shoulder, an upper arm, a forearm, a wrist, and an end effector.

(2) Motion capture system. Camera calibration and the creation of a global coordinate system that is parallel to the mobile platform's translational direction are both made easier by the motion capture system. The OptiTrack system (Motive Optical motion capture software., 2023) is the motion capture system used in this paper.

(3) Camera system. The camera system consists of two industrial cameras, which capture the operational status of the teleoperation power manipulator from two different angles. Table 1 provides the specific parameters of the cameras, including a focal length of 16 mm, a distortion rate of <0.2%, and a resolution of 5 million pixels.

**Table 1:** OPT-CC500-GM-04 camera parameters.

**Parameter type**	**Parameter value**
Data interface	GigE
Resolution	2,448(H)^*^2,048(V)
Chip size	2/3”
Maximum frame rate	30 fps
Pixel size	3.45 μm
Exposure time	34 μs-1 s
Optical interface	C
Size	29 mm x 29 mm x 42 mm

(4) Image processing module. The function of the image processing module is to locate the teleoperation power manipulator in a complex environment through the target detection algorithm, send the relevant information to the key points detection network for pose estimation, and input the pose information into the teleoperation power manipulator display module. The angles of the rotation joints of the teleoperation power manipulator are calculated based on the angles between the vectors formed by every two key points (O'Donovan et al., 2006). The translational joints are located by determining the translational distances of the key points in the 3D space through multiview-based triangulation (Zeng et al., 1999). The target detection and pose estimation methods of this module are the main research contents of this paper.

(5) The teleoperation power manipulator display module. The module was developed using Python and the V-REP Robot Simulator (Liu et al., 2017). To accurately represent the real teleoperation power manipulator, a model was created in Solidworks and subsequently imported into V-REP. The multiview teleoperation power manipulator pose estimation model is then utilized to continuously update the virtual teleoperation power manipulator's translational distances and pose information.

### 2.2. Preparation of the training dataset

To build a teleoperation power manipulator target detection model, the sample data for training the target detection model must be prepared first. The sample data are prepared in the following two steps:

(1) Acquisition of moving images of the teleoperation power manipulator

The image data are acquired mainly through the continuous acquisition of moving images of the teleoperation power manipulator from different angles through two cameras. To improve the robustness of the model, data were collected under different lighting conditions.

(2) Dataset labeling

**Figure 3:**
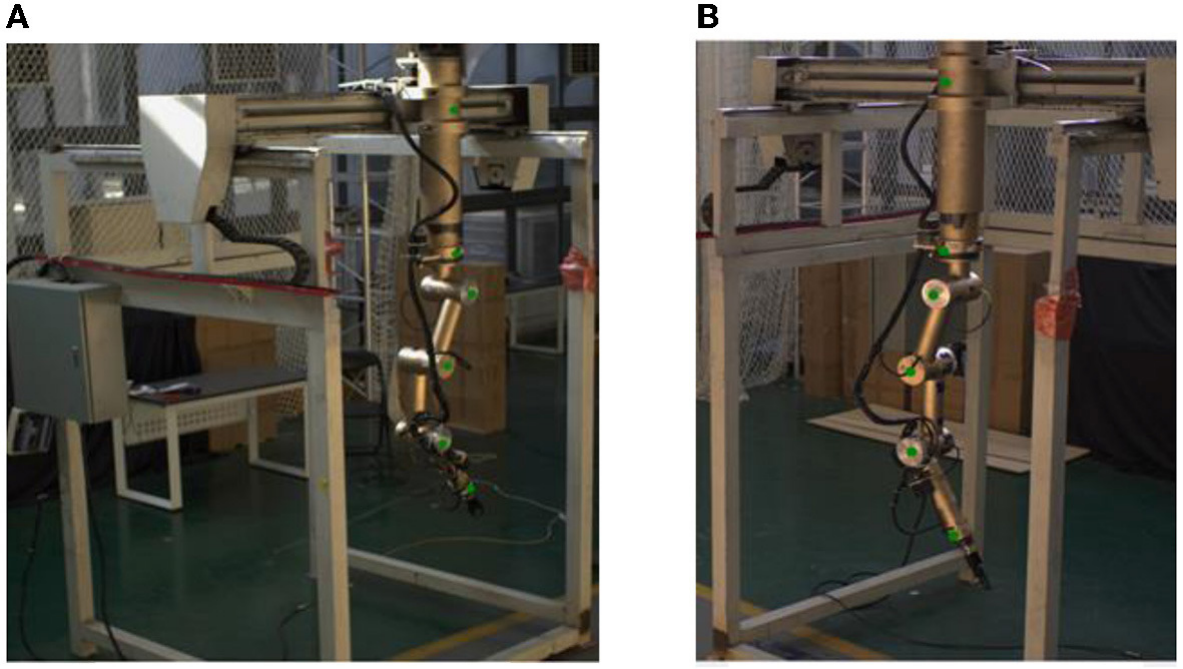
Keypoint labeling. **(A)** Camera(View)1. **(B)** Camera(View)2.

The key points (namely, the base, the shoulder, the upper arm, the forearm, and the wrist) of the teleoperation power manipulator are shown in Figure 3. The labeling tool LabelImg and the Visual Object Classes (VOC) Format are utilized in this paper. With reference to the MPII human pose estimation dataset (Simon et al., 2016), the files are labeled with the visibility and coordinates of the five key points. In addition, to improve the ability of the model to detect occluded key points, the slightly occluded key points were labeled and set to be visible. The different positions of the teleoperation power manipulator have different degrees of illumination during the operation. Color dithering is used to boost the robustness of the model to illumination, and random noise is added to the data to boost the model's robustness. The total number of samples generated was 4,000. The numbers of samples in the training set and the test set obtained after random allocation of the total samples were 3,600 and 400, respectively.

## 3. Dilated-FCOS method

**Figure 4:**
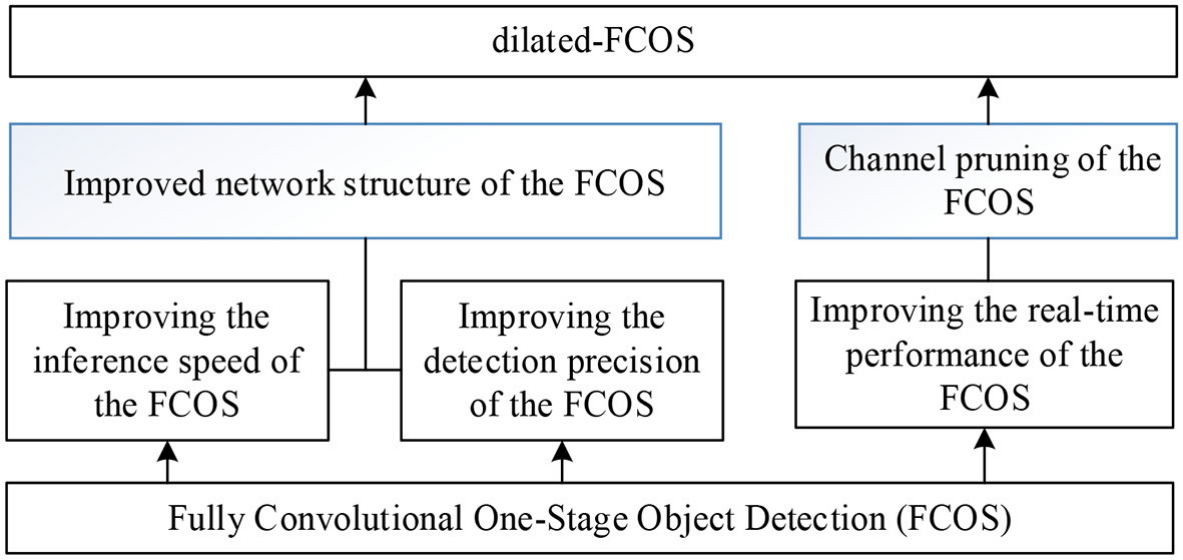
Dilated-FCOS method.

Fully Convolutional One-Stage Object Detection (FCOS) (Coppelia Robotics GmbH, 2022) is a fully convolutional anchor-free single-stage target detection algorithm. To suit the application of teleoperation power manipulator, a dilated-FCOS teleoperation power manipulator target detection method, is proposed. The structure of dilated-FCOS is shown in Figure 4.

(1) The improved network structure of the FCOS. According to the characteristics of the large target in teleoperation power manipulator detection, the FCOS network structure is modified to improve the detection precision, to reduce the time required for feature extraction, and to increase the model inference speed.

(2) Channel pruning of the FCOS. The FCOS target detection model's backbone network (darknet19) was optimized with the channel pruning algorithm to make it more precise and effective due to its high parameter redundancy and high computational overhead.

### 3.1. Method

#### 3.1.1. FCOS network

**Figure 5:**
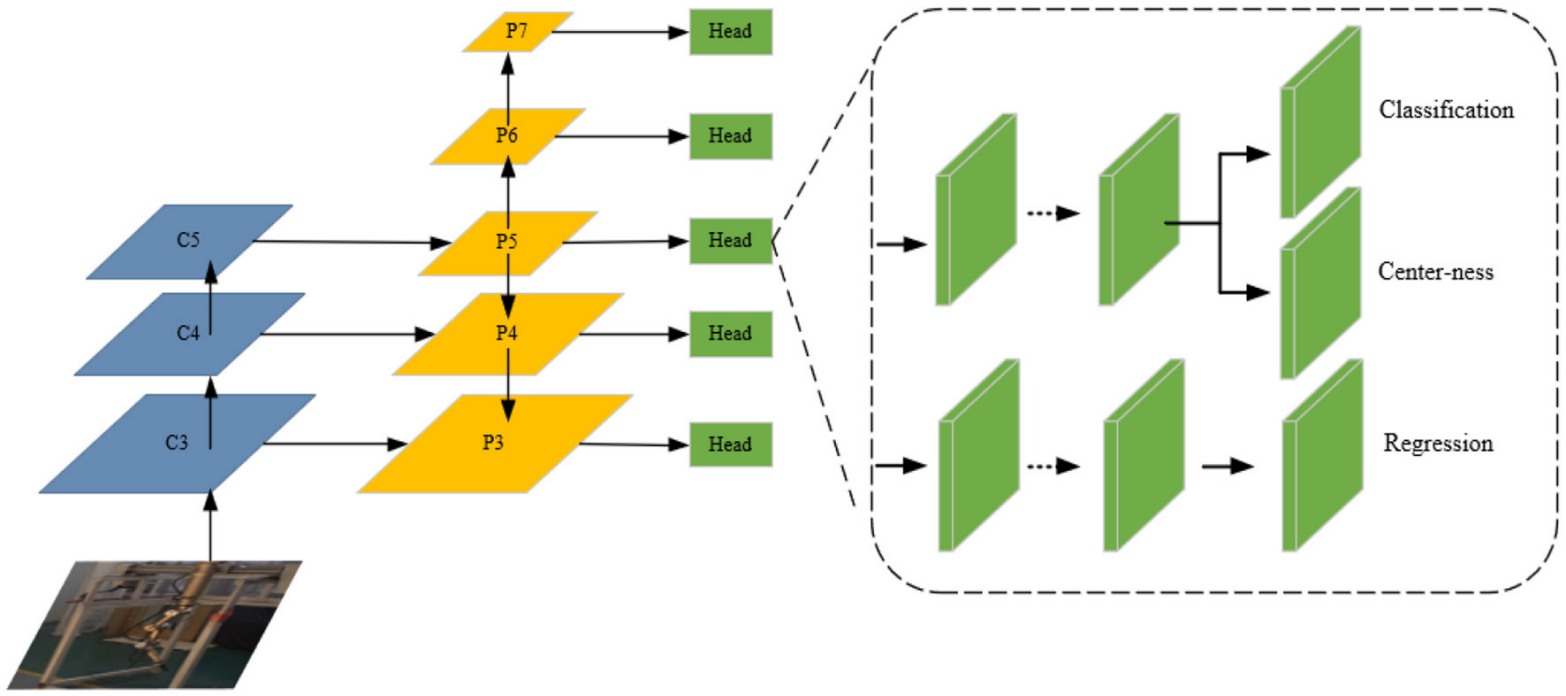
Network structure of the FCOS.

The structure of the FCOS network is shown in Figure 5. Darknet-19, the backbone network of FCOS (Andriluka et al., 2014), outputs three scale outputs (C3, C4, C5), and the feature pyramid outputs five scale outputs (P3, P4, P5, P6, P7). P3 is a high-resolution feature map with rich spatial information. P4 focuses on the detection of small targets. P5, P6, and P7 are low-resolution feature maps with rich semantic information, which focus on the detection of large and medium targets. The design concept of FCOS is divided into the following points:

(1) Pixel by pixel for the detection. Anchor-based algorithms often rely on artificially designing a significant number of anchor frames to enhance the recall rate. However, this approach introduces a challenge of imbalance between positive and negative samples during training, as the majority of anchor frames are negative samples. Additionally, the calculation complexity increases due to the intersection ratio between all anchor frames and boundary boxes during training. In contrast, FCOS is an anchor-free algorithm that avoids the use of anchor frames. Instead, it maps each feature point on the feature map to the original map and performs regression. By incorporating a larger number of positive samples, FCOS facilitates improved model training and leads to significant enhancements in the detector's performance.

(2) Multi-scale training strategy

**Figure 6:**
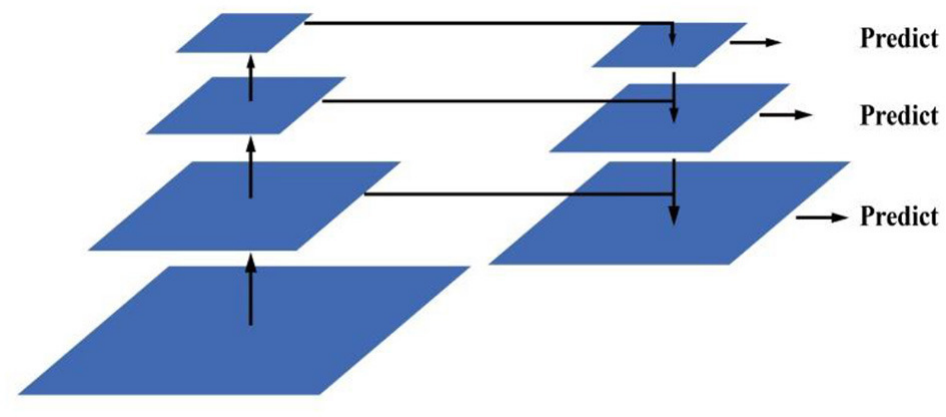
Feature pyramid networks.

Deep network has rich semantic information, that is, the output result is not affected by the position of the feature graph, which is suitable for classification task; the shallow feature has rich spatial information, that is, the output result changes according to the change of the features, which is suitable for regression task. Target detection requires both the regression of the target location and the target classification. To solve these two contradictory tasks simultaneously, FCOS adopts a feature pyramid structure to fuse the feature maps at different scales, so that the semantic information and spatial information between the different feature maps can complement each other. The feature pyramid network structure is shown in the Figure 6. The first part of the network is the path from the bottom, the backbone network, and the path is the lack of spatial information, and the features, adding the spatial information and semantic information of the feature map. In the third part, the lateral connection path adjusts the number of channels in the fusion to perform prediction and regression tasks. Integrating the information of different scales, the feature pyramid greatly improves the target detection accuracy of FCOS.

(3) Center confidence degree prediction

As shown in Figure 6, the central confidence degree is a branch increased in the prediction of each test head. The calculation of the central confidence is such as formulas (1). The detection box away from the central point is optimized by the cross entropy loss function. By combining the boundary box away from the object with the non-maximum suppression, the detection performance is significantly improved.


(1) $$\begin{array}{l} centerness=\sqrt{\frac{\min\left(l^{*},r^{*}\right)}{\max\left(l^{*},r^{*}\right)}\times \frac{\min\left(t^{*},b^{*}\right)}{\max\left(t^{*},b^{*}\right)}} \end{array}$$

where l^*^, r^*^, and b^*^ are the distance from the sampling point to the four sides of the boundary box.

#### 3.1.2. The improved network structure of the FCOS

**Figure 7:**
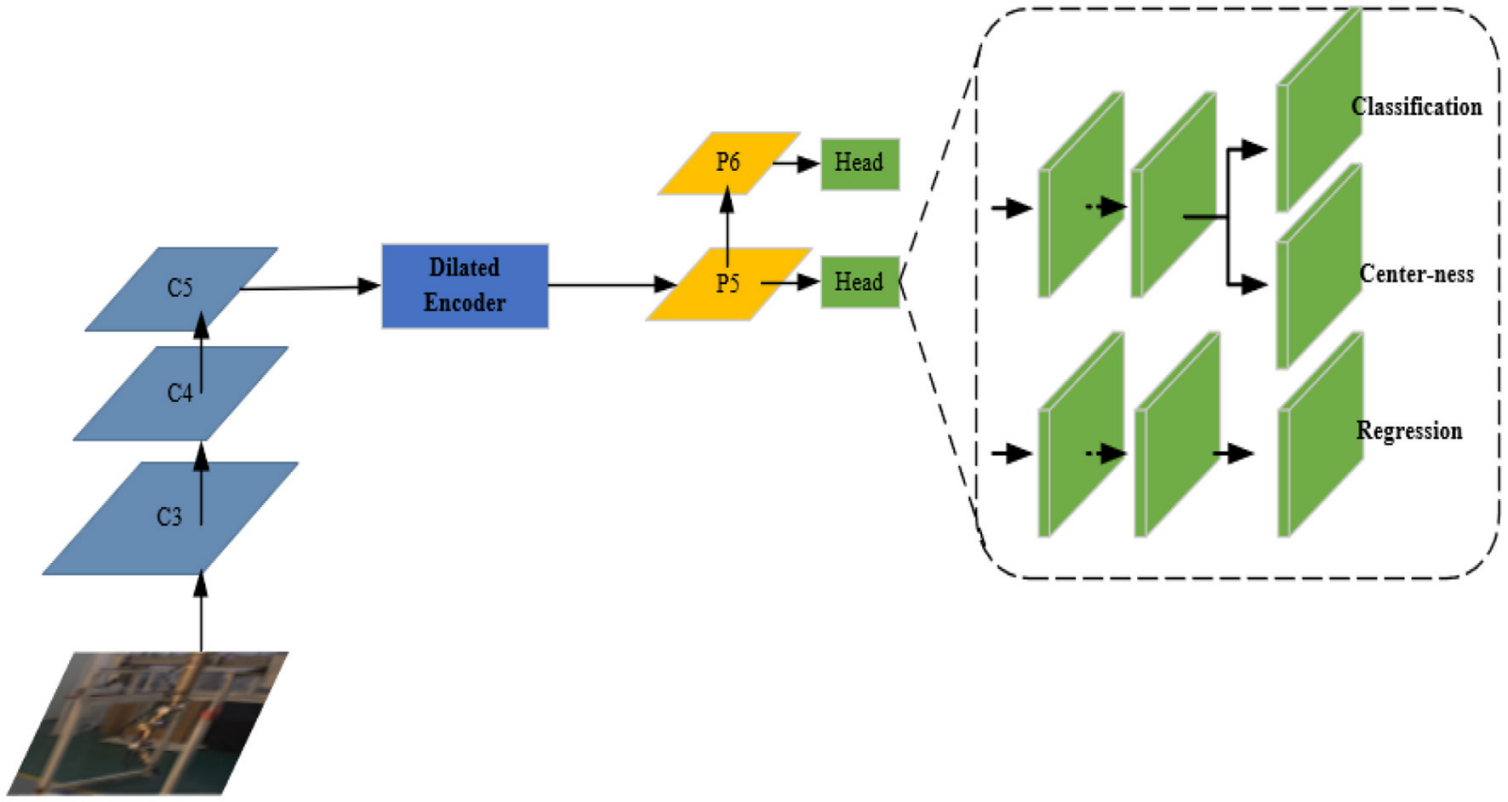
The improved network structure of the FCOS.

Figure 7 shows the improved network structure of the FCOS with two major improvements.

**Figure 8:**
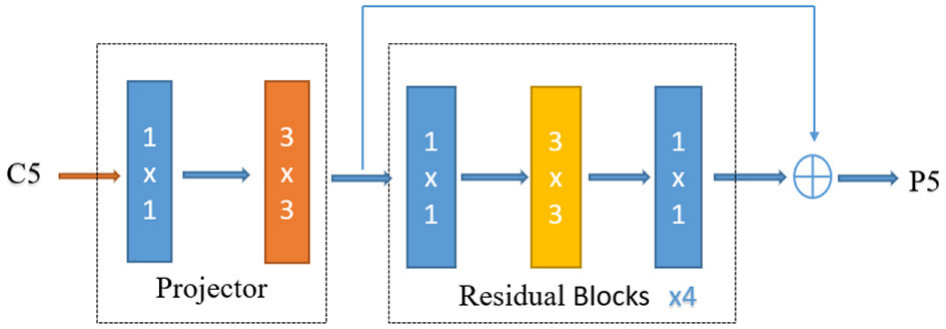
Dilated encoder.

(1) Improving the detection precision of the FCOS. Teleoperation power manipulator detection is a form of large target detection. Considering that the C5 feature layer has a limited detection scale range, a dilated encoder (Tian et al., 2020) is introduced to enrich the receptive field of the C5 feature layer by stacking continuous dilated residual units, and P6 is retained to improve the robustness of large target detection. The dilated encoder is shown in Figure 8. The first part of the encoder reduces the number of output channels through a 1 × 1 convolutional layer and then extracts semantic feature information through a 3 × 3 convolutional layer. The second part enlarges the receptive field through stacking continuous 3 × 3 dilated residual units with different dilation rates.

(2) Improving the inference speed of the model. Detecting the shallow features of small targets has very little effect on large targets such as teleoperation power manipulators. The shallow feature maps (P3, P4) are discarded here to improve the detection speed of the FCOS, and the P7 feature layer is discarded to improve the real-time performance of the network model. The improved network only performs the final classification, position regression, and central confidence interval prediction on the feature maps P5 and P6.

#### 3.1.3. Channel pruning of the FCOS

Channel pruning (Redmon and Farhadi, 2017) is a method that improves the real-time performance of a model by compressing the model. Through sparse training on the channel scaling factor, channel pruning leads to channel sparsification.

Adding a batch normalization (BN) layer (Chen et al., 2021) after the convolutional layer can achieve rapid convergence and better generalization performance. The calculation formulas of BN are as follows:


(2) $$\begin{array}{l} \begin{array}{c} \hat{Z}=\frac{{\text{Z}}_{in}-\mu }{\sqrt{\sigma^{2}+\varepsilon }}\\ {\;Z}_{out}=\gamma \hat{Z}+\beta \end{array} \end{array}$$

where Z_*in*_ is the input tensor, Z_*out*_ is the output tensor, **μ** is the vector of the mean value of the convolution result of each channel, σ is the vector of the variance of the convolution result, **ε** is a constant, **γ** is the learnable scaling factor in the BN layer, and **β** represents the learnable bias coefficient in the BN layer.

In Formula (2), when **γ** approaches 0, the effect of Z_*in*_ on Z_ou*t*_ is negligible. Here, **γ** is used as the scaling factor, and the parameter **γ** is penalized to save computational overhead and to avoid introducing unnecessary parameters.

The steps of channel pruning are as follows: (1) put all image data samples into the optimal model for sparse training; (2) sort the scaling factor **γ** of each BN layer; (3) prune the convolution layer corresponding to the scaling factor that has little effect on model performance; and (4) fine-tune the new model obtained by pruning to improve the detection performance of the network.

### 3.2. Test of dilated-FCOS

#### 3.2.1. Effectiveness test of the pre-trained model

**Figure 9:**
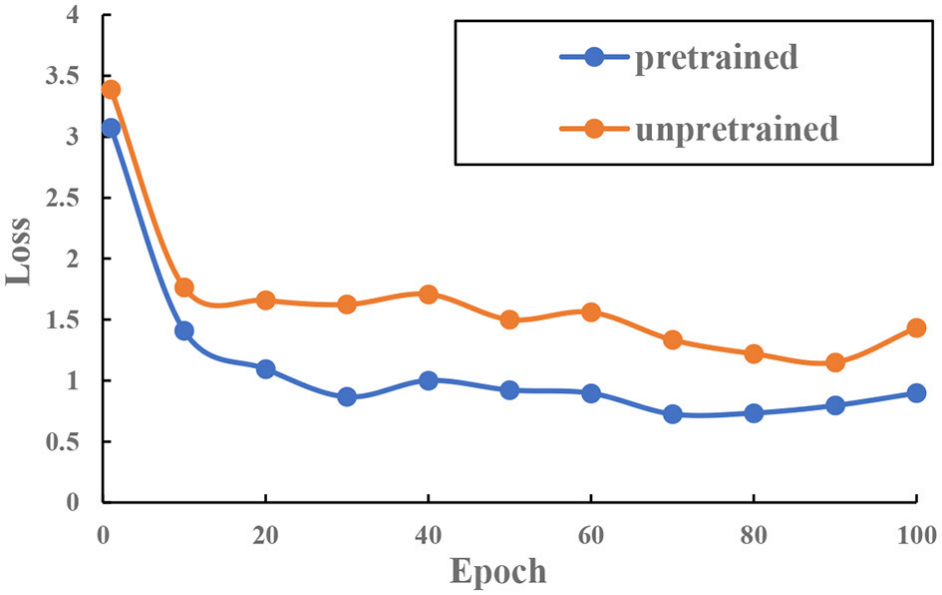
The loss–epoch curve.

Darknet19 is designed for ImageNet (Krizhevsky et al., 2012). Compared with the ImageNet dataset, the teleoperation power manipulator dataset is relatively small in size. Therefore, we first load and pre-train darknet19 on ImageNet to obtain the network weights to improve the network convergence speed. Two sets of experiments are set up to verify the effectiveness of the pre-trained model. Experiment 1 uses random weights to initialize the network, while Experiment 2 uses pre-trained weights on ImageNet to initialize the network. Both sets of experiments used the same learning strategy and optimization method. After 100 iterations, the loss curve was obtained, as shown in Figure 9. The results show that loading the pre-trained model can accelerate the model convergence.

#### 3.2.2. Performance test of target detection

FCOS, Faster-RCNN (Ren et al., 2016), and dilated-FCOS were used for the target detection performance test. In the experiment, the mean average precision (mAP) (Henderson and Ferrari, 2017) was used to measure the target detection performance of the model, and the inference time (ms) was used to measure the inference speed of the model. The intersection over union (IoU) threshold was set to 0.5, and a uniform image input size of 640 × 640 was used in all three models. The test results shown in Table 2 indicate that the dilated-FCOS is superior to the FCOS in both model precision and inference time.

**Table 2:** Performance comparison of different models.

**Model**	**mAP (%)**	**Inference time (ms)**
FCOS	93.78	31.59
Faster-RCNN	96.38	63.52
Dilated-FCOS	95.24	23.86

**Figure 10:**
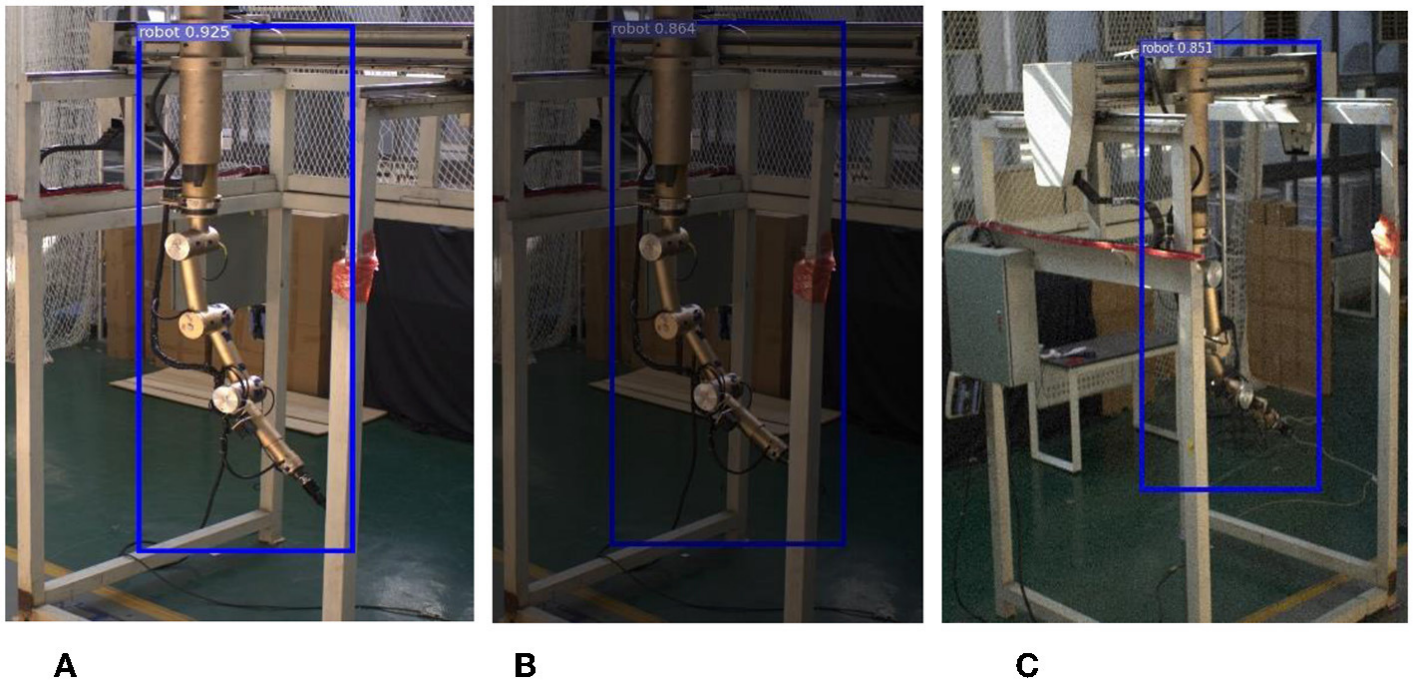
Results of network robustness. **(A)** Increase in brightness. **(B)** Decrease in brightness. **(C)** Adding noise.

To further test the robustness of the network, two sets of experiments were conducted in this study. In the first set, 640 x 640 images with color perturbations were used as inputs, while in the second set, images with noise interference were fed into the network. The partial experimental results, as shown in Figure 10, demonstrate that the network exhibits excellent anti-interference ability.

#### 3.2.3. Performance test of channel pruning

**Figure 11:**
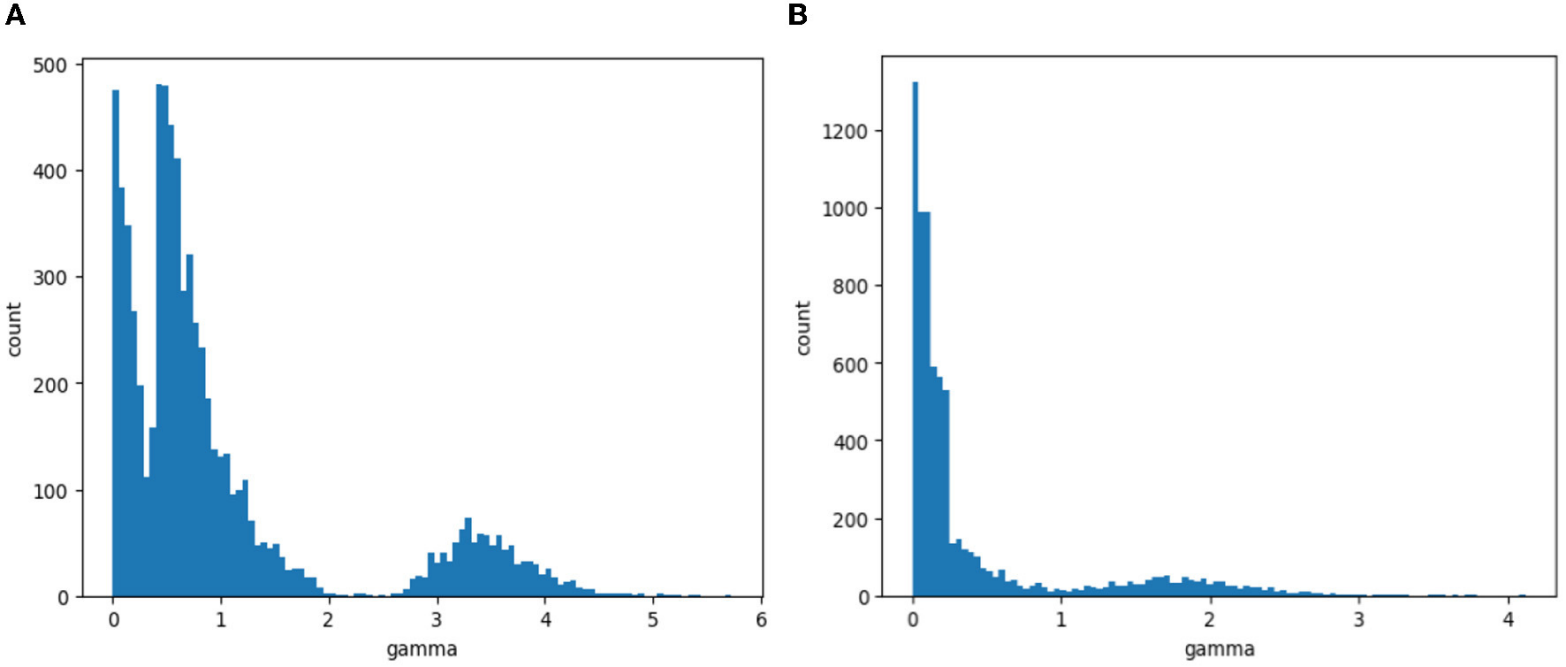
The distribution of γ at different λ values. **(A)** λ = 2. **(B)** λ = 5.

The first step of channel pruning is sparse training and screening out the channel numbers that have little impact on the output result. It is necessary to set the sparsity coefficient λ. Figure 11 shows the distribution of the scaling factor γ at different λ values. It can be seen that when λ = 2, γ is sparsified, but the effect is not obvious; when λ = 5, γ is close to 0, and the effect is obvious. Since λ = 5 is effective in screening the channel number, λ = 5 is selected to complete the sparse training.

Table 3 compares the performances of the model on the teleoperation power manipulator test set under different pruning rates. The original model has an mAP of 95.24%, a params of 35.96 M, and an inference time of 23.86 s on RTX 2080Ti. When the pruning rate is set to 0.1, the precision of the model increases slightly. This indicates that a higher precision can be achieved with fewer model parameters by removing the number of redundant channels of the original model. When the pruning rate is 0.1–0.6, the average precision of the model generally shows a slow downward trend. When the pruning rate is 0.6, the precision of the model reaches 92.78%. When the pruning rate is 0.7, the precision is reduced to 86.79%. These results indicate that channel pruning can maintain the precision of the model within a certain range and will damage the precision of the model after exceeding a certain threshold.

**Table 3:** The results of channel pruning.

**Pruning ratio**	**mAP (%)**	**Params**	**Compression ratio**	**Inference time (Ms)**
0	95.24	35.96 M	1	23.86
0.1	95.58	32.20 M	1.11	22.93
0.2	94.46	28.84 M	1.24	21.14
0.3	94.12	25.85 M	1.39	20.08
0.4	93.79	23.28 M	1.54	19.46
0.5	93.51	21.10 M	1.70	18.38
0.6	92.78	19.32 M	1.86	17.22
0.7	86.79	17.94 M	2.00	17.14

**Figure 12:**
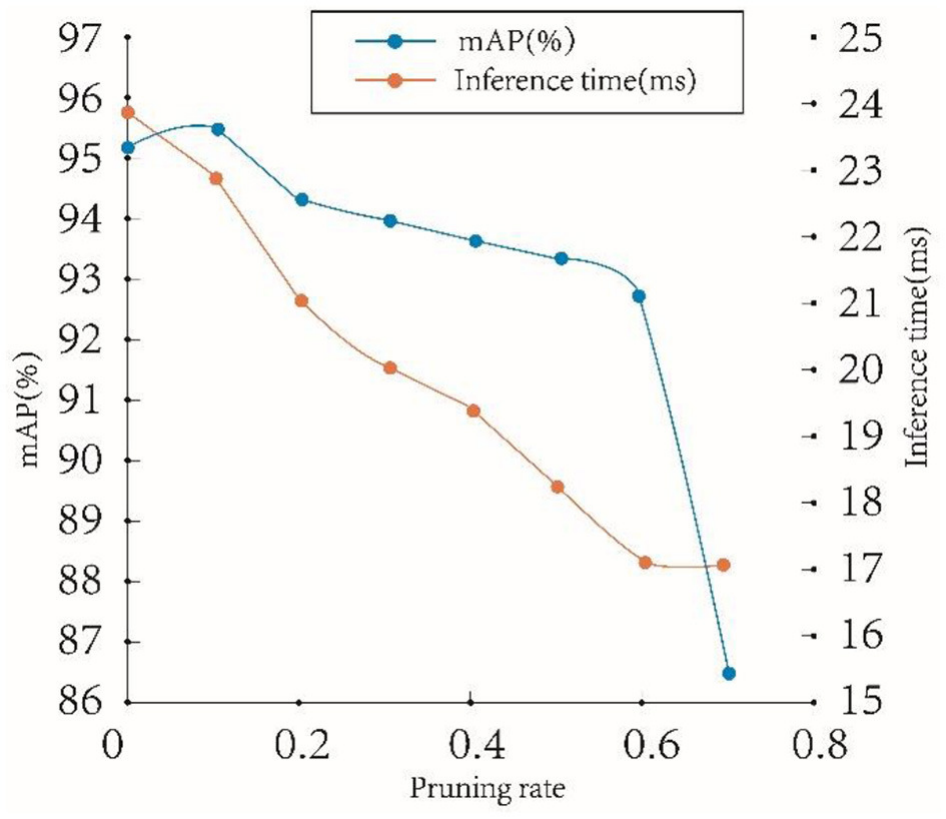
Different pruning rates of mAP and inference times.

Figure 12 shows the variation trend of the model precision and inference time on the teleoperation power manipulator dataset at different pruning rates. The model precision shows an upwards trend as the pruning rate increases from 0 to 0.1 and a gentle downward trend as the pruning rate increases from 0.3 to 0.6, while the inference time shows a more obvious downward trend as the pruning rate increases as the pruning rate increases, which indicates high model precision and small inference time delay at this time. When the pruning rate reaches 0.7, the precision decreases drastically, which indicates that pruning has severely damaged the precision of the model and has little effect on the optimization of the inference time. Therefore, the pruning rate is selected to be 0.5 in this paper to simultaneously achieve high precision and high inference speed.

## 4. Keypoint detection method

SimpleBaseline (Xiao et al., 2018) is a simple and efficient 2D human keypoint detection network composed of the backbone network ResNet (Szegedy et al., 2016) and three transposed convolutions that are responsible for upsampling to restore the resolution. In this paper, a SimpleBaseline-lite-based keypoint detection method for teleoperation power manipulators is established through two main steps: replacing ResNet with a lightweight backbone network to improve the real-time performance of the model; compressing the channels of transposed convolutions to improve the inference speed of the model.

### 4.1. Setting of model training parameters

**Figure 13:**
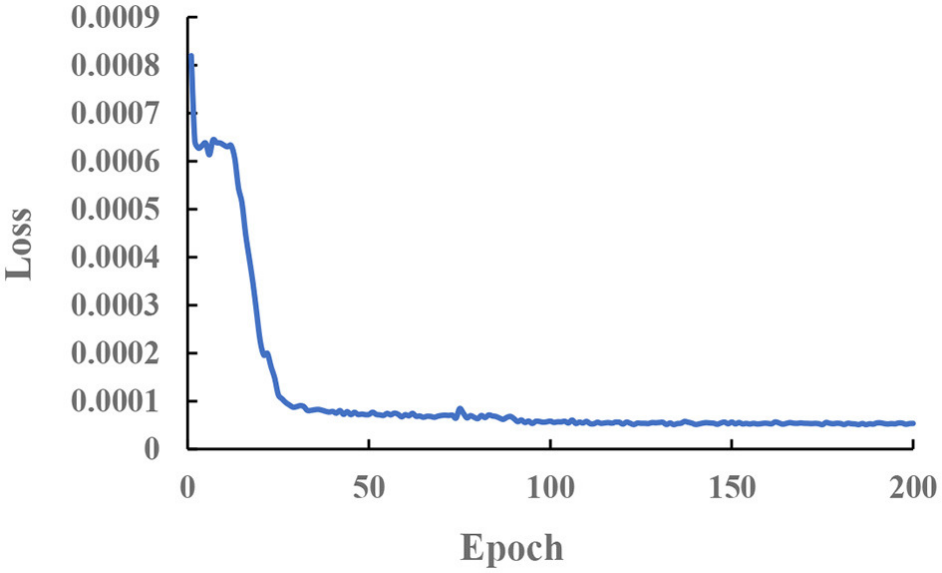
The diagram of loss.

In this test, the PyTorch framework is used for model training, and the number of iterations is 140 epochs. The warmup strategy is used to improve the convergence speed of the model. The learning rate increases as the number of iterations increases and reaches the initial learning rate. The initial learning rate of the optimizer Adam is set to 0.001, and when the number of iterations reach 50 epochs, its learning rate decreases by 10-fold. The loss function is shown in Figure 13. The model can complete the convergence in 70 epochs.

In this paper, the Percentage of Correct key points (PCK) (Xiao et al., 2018) is used to analyse the detection performance of the SimpleBaseline network. PCK is the percentage of the predicted key points with a normalized distance from the ground truth that falls within the set threshold. PCK is calculated using formula (3).


(3) $$\begin{array}{l} \begin{array}{l} \text{PCK=}\frac{\sum_{i}\delta \left(\sqrt{\left(x_{i}-\hat{x_{i}}\right)+\left(y_{i}-\hat{y_{i}}\right)},\varepsilon \right)}{\sum_{i}1}\\ \delta \left(a,\varepsilon \right)=\{\frac{1,a\leq \varepsilon }{0,a≻\varepsilon } \end{array} \end{array}$$

where (*x*_*i*_, *y*_*i*_) are the 2D coordinates of a keypoint, $\left({\hat{x}}_{i},{ŷ}_{i}\right)$ are the 2D coordinates of the keypoint predicted by the network, and ε is the pixel threshold.

**Figure 14:**
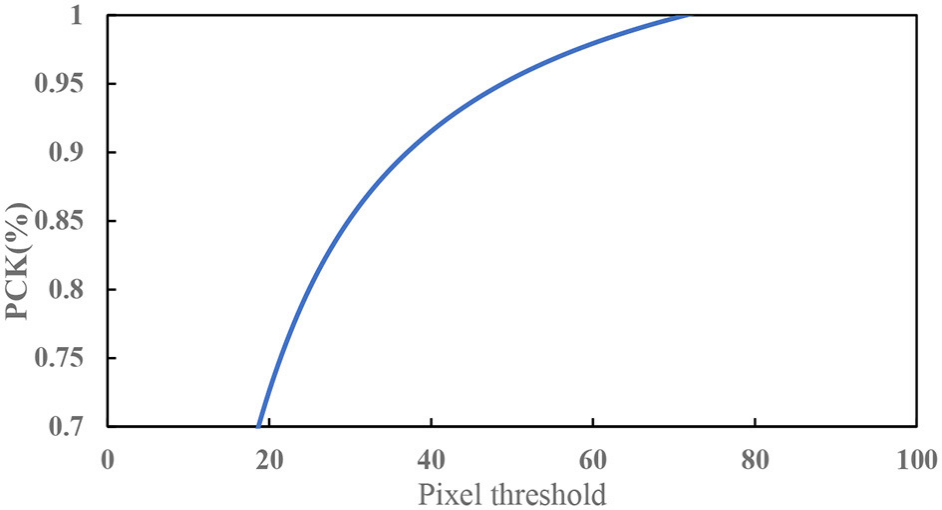
PCK under different pixel thresholds.

**Figure 15:**
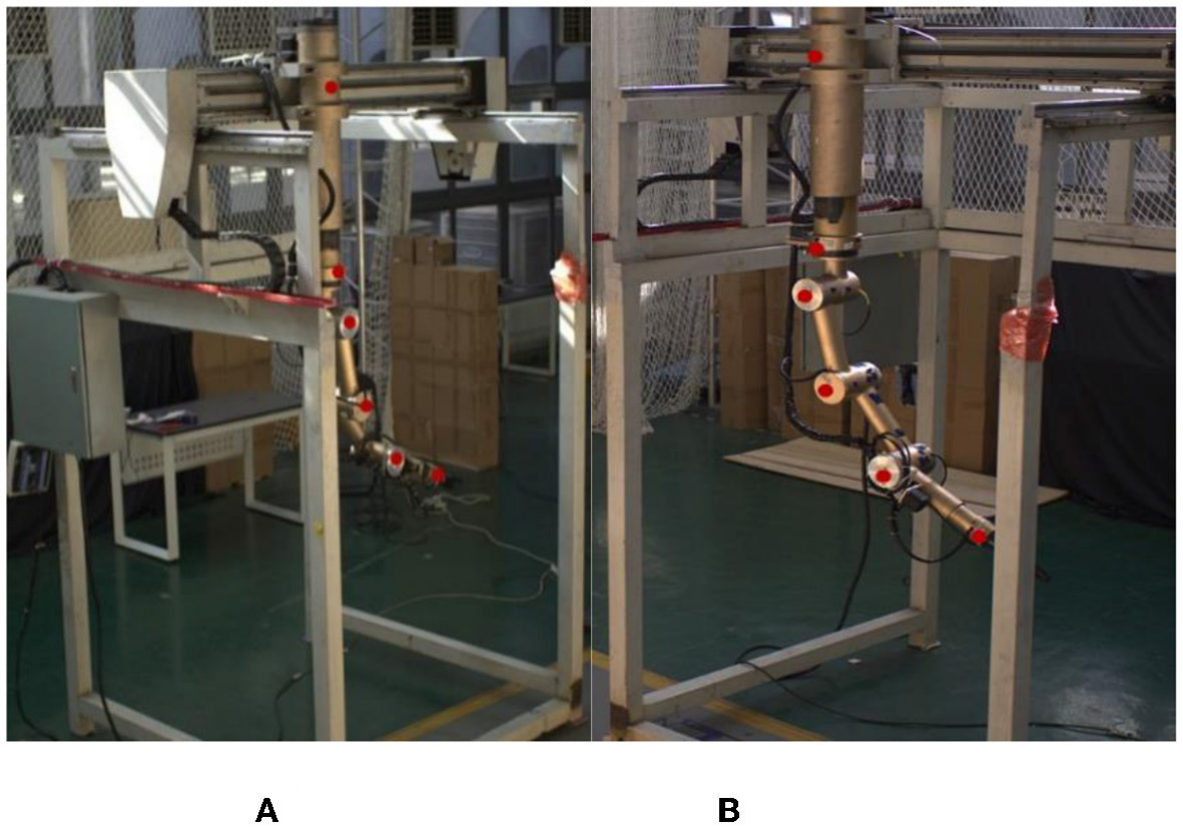
Visualization of prediction results on test sets. **(A)** Viewing angle 1. **(B)** Viewing angle 2.

Figure 14 shows the PCK of the 2D key points of the teleoperation power manipulator under different pixel thresholds. The experimental results show that the PCK reaches 91.5% under the pixel threshold of 40. Figure 15 shows the distribution of the key points predicted by the network, which indicates that the SimpleBaseline network has a good detection effect on the key points of the teleoperation power manipulator.

### 4.2. Test and selection of lightweight convolutional networks

**Figure 16:**
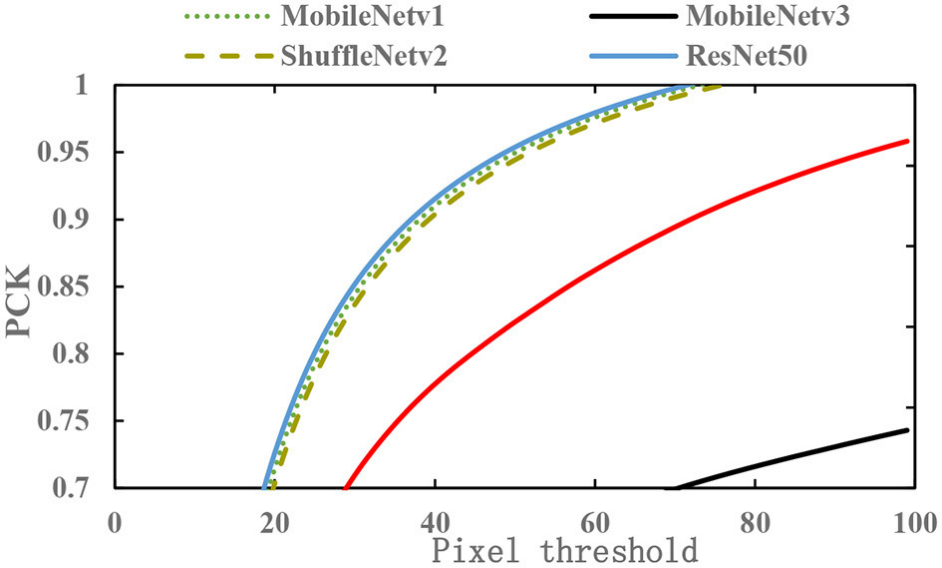
The PCK of different lightweight networks under different pixel thresholds.

The lightweight feature networks MobileNetv1 (Howard et al., 2017), MobileNetv2 (Liu et al., 2018), MobileNetv3 (Howard et al., 2020), and ShuffleNetv2 (Ma et al., 2018) are used to replace ResNet50 as the feature extraction network and are tested on the teleoperation power manipulator dataset. The results are shown in Figure 16.

Figure 16 shows that among the four types of lightweight networks, the sparsity coefficient λ of MobileNetv2 and that of MobileNetv3 have a relatively large decrease. Based on the analysis of the network structure, MobileNetv2 has many depthwise separable convolutions compared with MobileNetv1 and introduces an inverted residual structure to solve the problem of the deactivation of depthwise separable convolutions. However, compared with the traditional convolution, the depthwise separable convolution extracts less effective feature information, resulting in the lack of spatial localization information and affecting the model precision.

The detection of the 2D key points of teleoperation power manipulators requires the contextual information of the feature map, which requires rich spatial information. For low-dimensional feature maps, the greater the number of channels is, the more abundant the spatial information. Resnet50, MobileNetv1, and ShuffleNetv2 have many channels in the low-dimensional network layer and can achieve good results in the detection of key points of teleoperation power manipulators.

Table 4 shows the test performances of different feature extraction networks. The input image size for both training and testing is 800 × 160. The params of MobileNetv1-SimpleBaseline is only 27% of the original value, the computational complexity is reduced to 55% of the original value, and the inference time is reduced to 56% of the original value. In summary, the MobileNetv1-SimpleBaseline network is selected in this paper.

**Table 4:** The performance of the lightweight SimpleBaseline in the test set.

**Feature extraction network**	**Parameter**	**Computational complexity (GFLOPs)**	**Inference time (ms)**	**PCK@40 pixel(%)**
ShuffleNetv2	7.54 M	12.97	22.73	90.3
MobileNetv1	9.50 M	14.07	18.17	90.9
MobileNetv2	9.56 M	13.92	21.2	83.4
MobileNetv3	5.57 M	11.37	23.93	81.1
Resnet50	33.99 M	25.18	30.21	91.5

### 4.3. Pose distillation

The upsampling module of SimpleBaseline is composed of three transposed convolutions with 256 channels. As the resolution of the upsampling feature map increases, the computational overheads of the transposed convolutions also increase. Compared with ResNet50, MobileNetv1 has an inferior feature extraction performance and sparser input features of the transposed convolutions. Keeping the number of channels in MobileNetv1 the same as that in ResNet may cause model redundancy and reduce the inference speed.

In this paper, the model is optimized by compressing the number of channels in the transposed convolutional layer. The number of channels of the three transposed convolutions is set to 64 n, 32 n, and 16 n, respectively, i.e., 384, 192, and 96 (n is the number of key points, which is set to 6). After compressing the number of channels, the computational complexity is reduced to 1/3 of the original value, and the params is reduced to 2/3 of the original value.

Table 5 compares the performance of the MobileNetv1-SimpleBaseline after compression of the number of channels (SimpleBaseline-a) with the performance of the uncompressed network. After channel compression, the model redundancy is reduced, and the parameters and computational complexity are greatly reduced. Although the computational overhead is greatly reduced, and the detection time is only 64% of that of the original model, the detection precision has reached 94% of that of the original model. This result shows that there are still redundant parameters in the upsampling module of SimpleBaseline-a. Based on this network, a model with higher precision is designed through pose distillation in this paper.

**Table 5:** Comparison of the performances of SimpleBaseline-a and mobileNetv1-SimpleBaseline.

**Network**	**Parameter**	**Computational complexity (GFLOPs)**	**Inference time (ms)**	**PCK@40pixel(%)**
SimpleBaseline-a	8.75 M	6.49	14.73	87.4
MobileNetv1-SimpleBaseline	9.50 M	14.07	18.17	90.9

Pose distillation transfers the knowledge learned by a large network with good performance to a small network that is isomorphic or anti-isomorphic to the large network and compresses the model without significantly reducing the precision of the model (Hinton et al., 2014). The training process can be divided into two stages: training a powerful keypoint detection network as a teacher network and training a lightweight student model that simultaneously has high precision and high speed. The teacher model guides the student network to acquire high-level semantic information and strengthens the learning of the overall feature and spatial information by the student model.

Here, MobileNetv1-SimpleBaseline is selected as the teacher model, and SimpleBaseline-a is selected as the student model. The experiment is based on the PyTorch 1.5.1-GPU framework, the experimental operating system is Ubuntu 18.04, and the CUDA version is 10.2. The resolution of the network input image is 800 × 160, the initial learning rate is set to 0.001, the Adam optimizer is used, the batch size is set to 16, the momentum is set to 0.9, and the number of iterations is set to 140. The results are shown in Table 6.

**Table 6:** Comparison of model performance after distillation.

**Network**	**Parameter**	**Computational complexity (GFLOPs)**	**Inference time (ms)**	**PCK@40pixel(%)**
SimpleBaseline-lite	8.75 M	6.49	14.78	89.4
SimpleBaseline-a	8.75 M	6.49	14.73	87.4
MobileNetv1-SimpleBaseline	9.50 M	14.07	18.17	90.9

Table 6 shows that the model precision of the student model after pose distillation was improved by 2%, but the parameters, computational complexity, and inference speed did not change. The results show that pose distillation can improve the detection precision of the key points of the teleoperation power manipulator.

**Figure 17:**
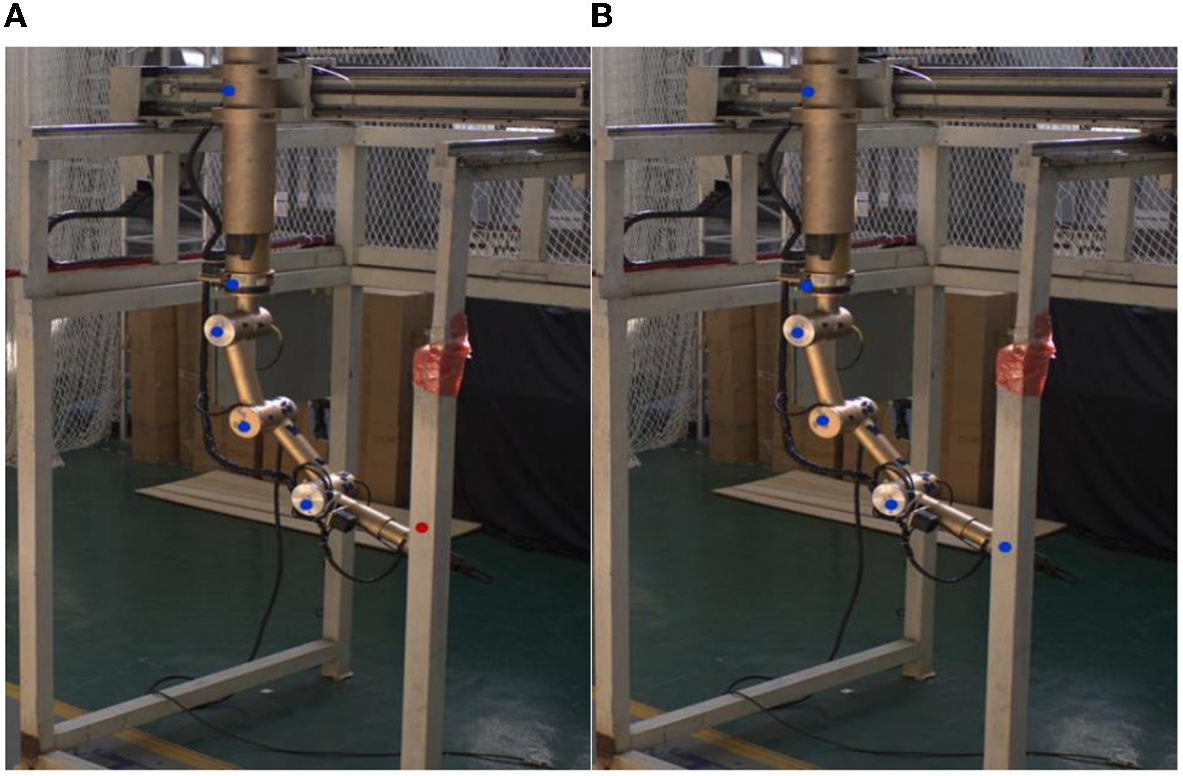
Visualization of model prediction results. **(A)** Before the “tutoring” by the teacher model. **(B)** After the “tutoring” by the teacher model.

The effectiveness of pose distillation is further illustrated by the visualized images in this paragraph. Figure 17 shows the predictions of the original student model (SimpleBaseline-a) and the student model after pose distillation (SimpleBaseline-lite) and the labeled visualized images. Occlusion and self-occlusion will inevitably occur in the teleoperation power manipulator (Figure 17A). Some occluded key points reduced the ability of the student model to extract spatial feature information, so the student model cannot fully learn the knowledge between channels of the feature map and the knowledge between the feature maps, resulting in a large deviation between the prediction result and the labels, which is the main reason for the decrease in detection precision. After the “tutoring” by the teacher model, as shown in Figure 17B, the student model has an enhanced ability to extract difficult-to-extract feature information because the teacher model can give the student model extra supervision due to its excellent ability to extract global spatial information. Pose distillation has improved the ability of the student model to detect the key points of the teleoperation power manipulator.

## 5. Experiment

In this section, we selected 10 arbitrary pose images of the teleoperation power manipulator during its operation. Simultaneously, we recorded the readings from the demonstrator of the teleoperation power manipulator. These demonstrator readings serve as the true values for our measurements. Our measurement objectives encompass seven evaluation objects: the translational distance along the x-axis, the translational distance along the y-axis, the translational distance of the shoulder, the rotation angle of the upper arm, the rotation angle of the forearm, the rotation angle of the wrist, and the translational distance of the wrist. To assess the accuracy of our measurements, we utilized the errors associated with each evaluation object in every image as our evaluation indicators. In Experiment 1, the improved dilated-FCOS and SimpleBaseline-lite were used for pose estimation of the teleoperation power manipulator. In Experiment 2, the FCOS and SimpleBaseline were used to initialize the network with training weights through the same optimization method.

The pose estimation performances of different algorithms are shown in Table 7. The improved dilated-FCOS + SimpleBaseline-lite algorithm is superior to the FCOS + SimpleBaseline algorithm in some tasks, such as translation along the x-axis, translation of the shoulder, the rotation angle of the upper arm, and translation of the wrist, because the improved dilated-FCOS achieves the stable detection of the position of the teleoperation power manipulator by introducing a dilated encoder based on the characteristics of the teleoperation power manipulator and thus lays a good foundation for the subsequent pose estimation task. Other tasks show no significant differences between the two algorithms, which indicates that model weight reduction and pose distillation of SimpleBaseline have not significantly affected the model precision. However, in terms of computational speed, the average frame rate of the improved dilated-FCOS + SimpleBaseline-lite algorithm reaches 5.8 fps, while that of the original FCOS + SimpleBaseline-lite algorithm reaches ~4.3 fps, which is 74% of that of the former. The results show that the pose estimation algorithm proposed in this paper has better performance in the teleoperation power manipulator pose estimation task than the FCOS + SimpleBaseline algorithm.

**Table 7:** Teleoperation power manipulator pose estimation experiment.

**Error**	**Model**	

	**Dilated-FCOS** + **SimpleBaseline-lite**	**FCOS** + **SimpleBaseline**
Translational distance along the x-axis/cm	6.27	6.36
Translational distance along the y-axis/cm	6.31	6.25
Translational distance of the shoulder/cm	4.32	4.34
Rotation angle of the upper arm/°	0.63	0.67
Rotation angle of the forearm/°	0.53	0.52
Rotation angle of the wrist/°	0.56	0.52
Translational distance of the wrist/cm	4.31	4.35

## 6. Conclusion

In this paper, the camera-based methods for target detection and pose estimation of teleoperation power manipulator is studied. The dilated-FCOS algorithm is proposed based on the FCOS algorithm and the scale of the teleoperation power manipulator. The shallow feature maps (P3, P4) of FCOS are discarded here to improve the detection speed of the FCOS, and the P7 feature layer of FCOS is discarded to improve the real-time performance of the network model. Model pruning is used to improve the real-time performance of the dilated-FCOS teleoperation power manipulator target detection model. To improve the detection speed for the key points of the teleoperation power manipulator, MobileNetv1 was selected as the backbone network based on the study of the SimpleBaseline algorithm and the comparison between keypoint detection precision and model inference speed of different lightweight backbone networks. To further optimize the inference speed of the model, the upsampling module was subjected to channel compression and pose distillation.

Our future work is as follows:

(1) The paper employs a motion capture system that relies on hand-eye calibration and an extrinsic calibration method for industrial cameras to track the movement of a teleoperation power manipulator. However, it is important to note that the current motion capture system may not be easily applicable in general scenarios. As a suggestion for future research, it would be beneficial to explore calibration methods that provide better generality and higher accuracy, addressing the limitations of the current approach.

(2) Model training is a critical aspect of supervised deep learning, where the quantity of training samples plays a significant role. However, in regular practice, the amount of available data is often limited. To address this limitation, future work can explore the utilization of simulation data from various scenarios to enhance the generalization ability of the model.

## Data availability statement

The original contributions presented in the study are included in the article/supplementary material, further inquiries can be directed to the corresponding author.

## Author contributions

LX: conceptualization, methodology, software, investigation, formal analysis, and writing—original draft. JH: data curation and writing—original draft. YL: visualization, investigation, and review and editing. JG: conceptualization, funding acquisition, resources, supervision, and writing—review and editing. All authors contributed to the article and approved the submitted version.
